# Sketch of the Present State of Medicine in Portugal

**Published:** 1838-07

**Authors:** 


### SKETCH OF THE PRESENT STATE OF MEDICINE IN PORTUGAL.

The medical institutions of Portugal, for the last two centuries, have participated in the general decline of that unhappy country, and only those students who have visited foreign schools have kept pace with the progress of science of other nations. But now, under the present dynasty, the medical practitioners of Portugal exert their best efforts to acquire that comprehensive knowledge which is necessary for the adequate exercise of their profession; a change which has been happily effected by the more general diffusion of education, that has sprung from the enlarged knowledge and liberal principles of foreign institutions, which the banished patriots brought with them from exile.

Coimbra is the only university in Portugal where medicine is taught: it confers degrees in medicine and in surgery. It was founded in 1291, by King Dennis, and had at that time fifty professors. At Coimbra, as elsewhere, theoretical principles were those chiefly insisted on in medicine; and, even when a better system was adopted by other universities, Coimbra, in character with the inhabitants of the country, failed in emulating the improvements, and consequently fell into disrepute. There were also other causes, more directly lessening its importance, arising from the scanty facilities presented in the town for the study of anatomy, or any of the practical departments of medicine, and chiefly from the interference, both of professors and students, in the political disturbances of the day. These causes sufficiently explain its decline; and the recent proposal to transfer the medical school to Lisbon, if carried into effect, will prevent its ever reacquiring its former celebrity, at least as a school of medicine.

Besides the university of Coimbra, there are schools of surgery at Lisbon and at Oporto. These schools were established in 1824, by Don John VI.; but only since 1825 has any regular course of instruction been attempted. That in Lisbon is connected with the hospital of St. Joseph, and here the school of anatomy, and also of surgery, is excellent. Many eminent men have already commenced and finished their education under its teachers. The supply of subjects for anatomical and surgical purposes is most ample; and foreigners meet with every encouragement in prosecuting this important branch of study.

Before the establishment of the schools of Lisbon and Oporto, the great bulk of medical men in Portugal were very ignorant, knowing little of either anatomy or practical surgery: indeed, many surgeons were in practice who had never studied at any school. These men practised on the attestation of other surgeons, after an examination which was not conducted before professors or teachers, but before surgeons chosen in all parts of the country, as suited convenience, by the chief surgeon of the kingdom. Matters went even further than this; surgery being sometimes practised by men who had never received any authority at all for doing so: and of this class there are still some examples to be found.

Much is still wanting to perfect medical education in Portugal; and, indeed, the only plan of sustaining the stimulus now existing among the Portuguese medical men is by carrying into effect the design, which has been for some time entertained, of establishing a regular college at Lisbon, where every facility may be commanded for the furtherance of study.

The following is a list of the hospitals at present established in Lisbon:

| | |
|---|---|
| Hospital of St. Joseph . . . | 1000 beds. |
| Marine Hospital . . . . | 150 .. |
| Military Hospital, at the Castle . . | 300 .. |
| at Belem . . | 100 .. |
| Hospital of St. Lazarus . . . | 30 .. |
| Victoria (for aged Women) . | 12 .. |

In addition to the foregoing, it must be understood that each religious house has an hospital attached to it.

Of the above, that of St. Joseph is the only civil hospital. The number of patients in this establishment, it is obvious, gives ample scope for clinical teaching and anatomical research; but the city of Lisbon, if under proper regulations, might present a field far more extensive than exists at present for these studies; for it is notorious that the demand for admission into the hospitals is greater than can be complied with: even under present circumstances, the opportunities are great. The number of patients admitted into St. Joseph's, during the spring and summer quarters of 1835, (six months,) were 14,066; of which number, 2,077 died: and, during the autumnal quarter of 1836, we perceive, from a report in the Lisbon Medical Journal, that the numbers were—admitted, 3,725; cured, 3,281; died, 444.

The arrangements of the Foundling Hospital are much complained of; and when we consider that on such institutions the population of the city depends in a considerable degree, we are not surprised to find that this subject is much talked of at present in Portugal. There is no well-regulated Lunatic Asylum; and the classification of patients, if understood at all, is not practised in Portugal.

In Portugal there is no prevailing system of medicine which can be termed national: the mode of study, the favorite theories, and the practice, are, in general, more similar to the French than those of any other. The doctrines of Stahl, Hoffmann, Boerhaave, and Cullen, have alike had their day in Portugal, as in other European countries. The celebrity of Broussais, and the apparent clearness and simplicity of his doctrines, soon led the great body of Portuguese medical men to become his followers. They still continue to be so; but there were, from the first, a good many physicians, who, while they adopted many of his views, yielded to none of his extravagance, and followed, and still follow, his tenets with a reasonable and prudent moderation. This will appear from the following extract from a recent essay by Dr. F. A. Barral; which will serve, at the same time, to give a general outline of the ordinary course of practice in Portugal.

"We consider fevers as local irritations of different organs; we devote much attention to the state of the stomach and intestines previous to the administration of medicines; and we do not neglect the prudent use of emetics and purgatives in functional disorders and subacute irritations of organs; the advantages of which have been proved by time and experience. We employ bloodletting and the antiphlogistic treatment with sufficient frequency, but we are not reduced to this mode of treatment only—despising all others which physicians, in all times, have represented as beneficial. So also, in obscure cases, we employ substances of less evident power, but which, at various periods, have had ascribed to them many and different properties by practical physicians. Camphor, calomel, tartar emetic, and all the antimonial preparations, have been much employed, and with good results, in inflammatory affections, after the most violent symptoms have been combated by more direct antiphlogistic and revulsive means; when the disease seems to resist these means, and when the digestive apparatus permits their exhibition. The use of tartar emetic in acute pneumonia, in repeated minute doses, was known and approved of in Portugal, long before the period at which it was introduced first into Italy, and more recently into France. The homœopathic doctrine has been known in Portugal for the last twelve years, but has not been followed with much enthusiasm."

The employment of the bark of the pomegranate root in tænia is claimed as a national discovery, although, we believe, it has been employed for ages in India for this purpose.

Portugal abounds in mineral waters, chiefly chalybeate and sulphureous; but as yet we have no accurate scientific account of them. The "Caldas da Rainha," the most celebrated, are near Lisbon, and are sulphureous.

In Surgery, particularly operative surgery, the Portuguese are very well advanced; the opportunities during the late French war, and the recent civil contest, gave them ample practical experience, which has not been thrown away. Their military surgical establishments are, however, still at a low ebb; and this inefficient state was much felt, and much to be deplored, during the war.

A striking proof of the impulse which has, of late years, been given to medicine in Portugal is the greatly increased activity of the medical press. In the summer of 1835, two medical journals made their appearance, and have continued to be regularly published since that time. They both originate from Medical Societies, and they are both very creditable productions. *The Society of the Medical Sciences of Lisbon* (Sociedade das Sciencias Medicas de Lisboa,) was instituted in May 1835, and consists of physicians, surgeons, and apothecaries, foreign as well as native. *The Pharmaceutical Society of Lisbon* (Sociedade Pharmaceutica de Lisboa,) was instituted in July of the same year, and consists also of members from all the classes of the profession; although its views are more especially directed to objects of pharmacy, natural history, and therapeutics. The *Jornal da Sociedade das Sciencias Medicas de Lisboa* appears monthly: it is of the octavo size, and each number contains four sheets of letterpress. The contents are—copious reports from the Lisbon hospitals, an account of the business transacted at the meetings of the Society whence it emanates, original papers of considerable interest, extracts from foreign journals, and notices of foreign publications. In our last Number we have given some extracts from this journal, and hope to be indebted to its pages for matter still more interesting.

The *Jornal da Sociedade Pharmaceutica de Lisboa* is conducted on the same general plan, but contains much less information on subjects of general interest to the practitioner. It seems, however, well conducted, and indicates an active state of the Association from which it springs.

Remembering, as we do, what Lisbon was some twenty-five years ago,—a sink of filth, ignorance, sloth, and superstition, and the medical profession partaking largely of these attributes of the general population,—we feel convinced, on the mere evidence furnished by these journals, that no country has, within a short period, made more rapid strides in real civilization than Portugal. In these journals we discover no indication that Portugal yet possesses an original medical literature; nor was this to be expected. Such a literature is necessarily a plant of slow growth, and requires a civilization of some duration. We find, however, sufficient indications in them of a vigilant observation of what is passing abroad, and of an intellectual exertion at home, which will in time produce indigenous fruit. The physiological system of Broussais, as we have already remarked, is manifestly the reigning doctrine in this country: it mingles in almost all the reasonings of their writers, and imparts a tinge to the nomenclature of their reports. We observe, however, that some of the original communications in the journals display a strong spirit of independent observation, and that the writers (as stated by Senor Barral) rather cull from the doctrines of Broussais what they deem to be valuable than fulfil implicitly all his mandates.*

* For the above sketch we are principally indebted to the kindness of Dr. P. S. Fraser.—Eds.

